# Malignant Peritoneal Mesothelioma: A Rare Cause of Ascites

**DOI:** 10.1177/2324709618807506

**Published:** 2018-10-16

**Authors:** Abdisamad M. Ibrahim, Mohammad Al-Akchar, Zainab Obaidi, Hamid Al-Johany

**Affiliations:** 1 Southern Illinois University, Springfield, IL, USA; 2 Johns Hopkins University, Baltimore, MD, USA

**Keywords:** peritoneal mesothelioma, mesothelioma, asbestos exposure

## Abstract

Malignant peritoneal mesothelioma (MPM) is a rare diagnosis that presents with difficulties in diagnosis and management. This article reports a case of an 88-year-old male who presented with a 2-week history of abdominal distention and bloating. He worked at an insulation production factory between the ages of 23 and 25 years with presumed asbestos exposure. On the computed tomography scan of the abdomen/pelvis, the patient was found to have diffuse omental, peritoneal, and mesenteric nodularity with moderate to large ascites. Omental biopsy revealed MPM. The overall prognosis of MPM remains poor, with a median survival time of 12 months at the time of diagnosis. Treatment modalities offered in the United States include chemotherapy alone, cytoreductive surgery alone, or cytoreductive surgery/chemotherapy combination.

## Introduction

Malignant peritoneal mesothelioma (MPM) is a rare and aggressive disease that arises from the lining mesothelial cells of the peritoneum. The incidence of MPM is one in a million, and in the United States, it accounts for 10% to 15% of all cases of mesothelioma.^1,2^ There is a strong link between asbestos exposure and development of MPM, with approximately 50% of reported cases having asbestos exposure. The latency period between asbestos exposure and development of mesothelioma is approximately 40 to 45 years; thus, diagnosis can be challenging at the time of presentation.^2^ This article reports an extremely rare case of MPM, epithelioid subtype, which was complicated by ascites.

## Case Presentation

**Figure 1.**
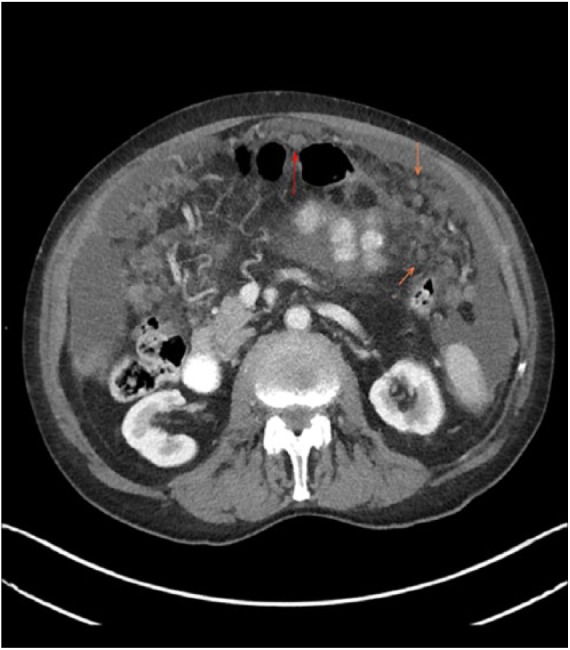
Computed tomography scan of the abdomen showing peritoneal nodularity.

An 88-year-old male was admitted to the medical floor with 2-week history of abdominal distention and bloating. The patient reported associated decreased appetite, early satiety, and generalized weakness. He was actively working as a part-time barber for the past 55 years. Prior to that, he worked at an insulation production factory between the ages of 23 and 25 years with presumed asbestos exposure. Additional exposure history significant for 10 pack-year smoking (1 pack × 10 years) and significant passive smoking exposure. Physical examination was notable for distended abdomen with mild tenderness to deep palpation in all quadrants. There was no rebound tenderness or guarding. Fluid wave test was positive, and he had lower extremity edema. Initial laboratory workup was unremarkable, except for low serum albumin (Table 1). Computed tomography (CT) scan of the abdomen/pelvis with contrast showed diffuse omental, peritoneal, and mesenteric nodularity with moderate to large ascites (Figure 1). Given these new findings, workup was directed to look for the primary malignancy. CT chest with contrast was done, which was negative for primary lung malignancy. However, CT chest with contrast showed bilateral pleural plaques indicating prior asbestos exposure. Esophagogastroduodenoscopy and colonoscopy were unremarkable. Tumor markers CEA, PSA, CA 19-9, and AFP were also within normal limits. Therapeutic and diagnostic paracentesis were done, which yielded 2.5 liters of blood-tinged fluid. Ascitic fluid analysis revealed the values shown in Table 2.

**Table 1.** Laboratory Evaluation.

Hemoglobin	14.6 g/dL
Hematocrit	44%
White blood cell	8.6 K/cumm
Platelets	314 K/cumm
Sodium	136 mmol/L
Potassium	4.5 mmol/L
Blood urea nitrogen	14 mg/dL
Creatinine	1.2 mg/dL
Alkaline phosphatase	50 IU/L
Aspartate aminotransferase	36 IU/L
Alanine aminotransferase	26 IU/L
Albumin	2.9 g/dL
Total protein	4.7 g/dL

**Table 2.** Ascitic Fluid Analysis.

Fluid white blood cells	720/cumm
Fluid red blood cells	60 075/cumm
Fluid neutrophils	10%
Fluid lymphocytes	64%
Fluid monocytes	26%
Fluid macrophages	Moderate
Fluid mesothelial cells	Moderate
Fluid glucose	73 mg/dL
Fluid pH	7.0
Fluid protein	3.4 g/dL
Fluid albumin	2.2 g/dL

**Figure 2.**
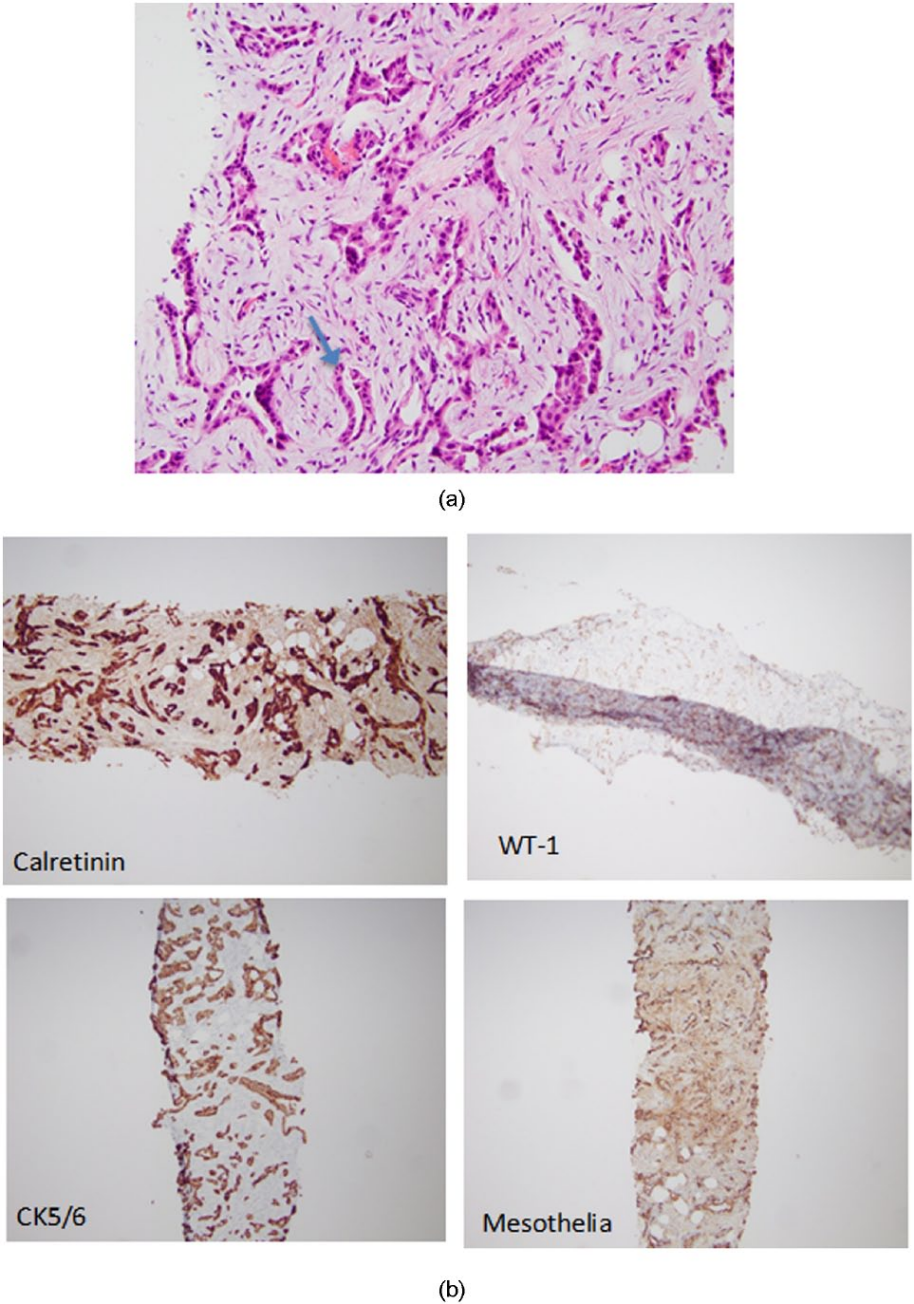
(a) Omental biopsy showing malignant mesothelial cells with pseudoglandular formation (hematoxylin-eosin stain, 40×). (b) Tumor cells are positive for calretinin, WT-1, CK5/6, and mesothelia, confirming diagnosis of malignant peritoneal mesothelioma.

Serum-ascites albumin gradient was calculated at <0.7, indicating ascites not associated with portal hypertension. Given the findings of the fluid analysis and CT abdomen, the patient underwent ultrasound-guided omental biopsy. Tumor cells were positive for calretinin, WT-1, CK5/6, and mesothelia (Figure 2) confirming the diagnosis of MPM, epithelioid subtype. Given the patient’s advanced age and his medical comorbidities (coronary artery disease and hypertension), the patient was deemed not a candidate for cytoreductive surgery (CRS) or platinum-based chemotherapy. With the poor prognosis of MPM in mind, the patient opted not to pursue further treatment and decided to go home with home hospice. Palliative peritoneal catheter was placed, and the patient was discharged home with home hospice care.

## Discussion

Mesothelioma is a rare malignant neoplasm that arises from the cell lining of the serosal surfaces.^1^ It commonly arises from the pleural serosal surface, more often than the peritoneum. This is the case in 10% to 15% of mesotheliomas, as is in our patient.^1,2^ As the case in other forms of mesotheliomas, the cumulative asbestos exposure remains the leading cause of MPM.^3^ Other etiologies include para-exposure, such as laundering the clothes of an exposed person, therapeutic irradiation, chronic inflammatory peritonitis, and simian virus-40.^3^

MPM is a challenging entity. First, diagnosis can be difficult to establish given the nonspecific symptoms at the time of presentation, resulting in delayed diagnosis. The estimated mean time to diagnosis is 122 days.^4^ The second challenge, as illustrated before, is that not all patients have a history of asbestos exposure. Therefore, when faced with abdominal pain and/or abdominal distention/ascites, which are the 2 important manifestations of MPM, especially in the history of asbestos exposure, it is essential to seek further imaging. CT scan of the abdomen can help make the diagnosis and usually can show diffuse peritoneal masses invading the omentum, pleural thickening, nodularity, and/or omental caking.^3,4^ Diagnosis should be confirmed through omental biopsy.

The overall prognosis of MPM remains poor, with a median survival time of 12 months at time of diagnosis.^3^ Treatment modalities offered in the United States include chemotherapy alone, CRS alone, and CRS/chemotherapy combination. In a recently published study, poor prognostic variables included advanced age, male gender, uninsured/Medicaid insurance, and sarcomatoid/biphasic histology.^5^ In addition, combined modality of treatment (CRS/chemotherapy) seems to be associated with the longest median survival, 61 months.^5^

In the index case, the patient had poor prognostic factors, such as male gender and advanced age. He also had multiple medical comorbidities, such as coronary artery disease and hypertension. Given the above-mentioned conditions, risks and benefits of CRS and chemotherapy were put into consideration. Our patient opted not to pursue further treatment and decided to go home with home hospice.

This case report highlights the importance of including MPM in the differential diagnosis of patients presenting with new ascites, especially with history of asbestos exposure. It is important to seek further diagnostic imaging including CT scan of the abdomen and biopsy of any suspicious lesions. Unfortunately, the prognosis remains dim in this condition.
